# Supplementary material for: Disrupting iron homeostasis can potentiate colistin activity and overcome colistin resistance mechanisms in Gram-Negative Bacteria
Source: Commun Biol. 2023 Sep 13;6:937. doi: 10.1038/s42003-023-05302-2 (PMC10499790; doi:10.1038/s42003-023-05302-2)
Supplement: Supplementary file 2 — Supplementary Information [file 42003_2023_5302_MOESM2_ESM.pdf]

Supplementary Information

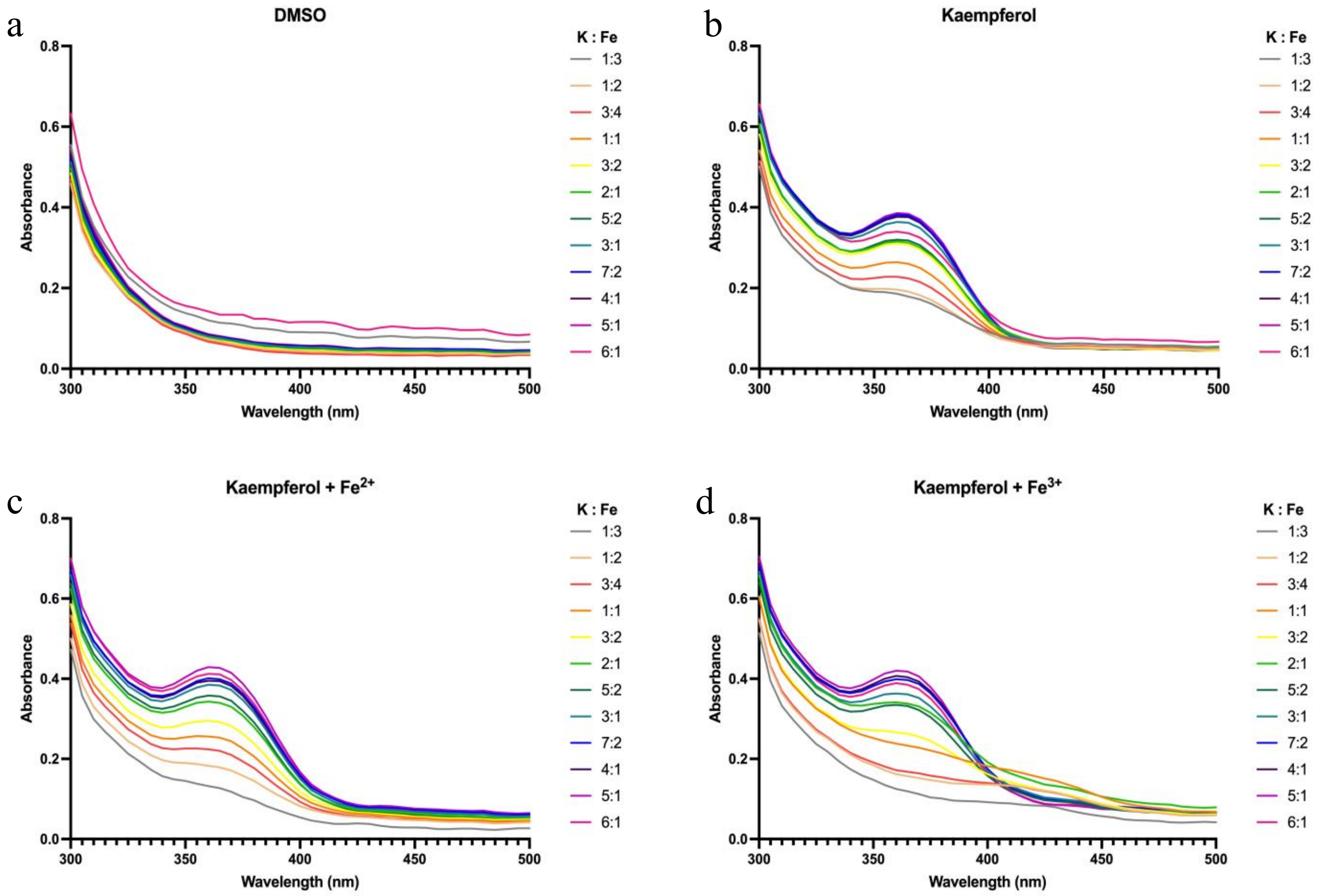

Supporting Information Figure S1. Kaempferol binds to Fe^{3+}. Absorption spectra in the 300-500 nm range of kaempferol (peak at ~ 360 nm) compared to the DMSO control (kaempferol carrier) and mixtures of kaempferol with either Fe^{2+} or Fe^{3+}. The kaempferol peak remained unaffected upon the addition of Fe^{2+}. However, after addition of Fe^{3+}, in the mixtures with the lowest kaempferol:Fe^{3+} ratio the kaempferol peak shifts to lower wavelengths, indicating that kaempferol specifically interacts with Fe^{3+}. One representative example out of three independent replicates is shown. For each kaempferol:iron proportion, the respective concentrations, in mM units, were: 1:3 -> 0.025:0.075, 1:2 -> 0.033:0.067, 3:4 -> 0.043:0.057, 1:1 -> 0.05:0.05, 3:2 -> 0.06:0.04, 2:1 -> 0.067:0.033, 5:2 -> 0.071:0.029, 3:1 -> 0.075:0.025, 7:2 -> 0.078:0.022, 4:1 -> 0.08:0.02, 5:1 -> 0.017:0.083, 6:1 -> 0.086:0.014.

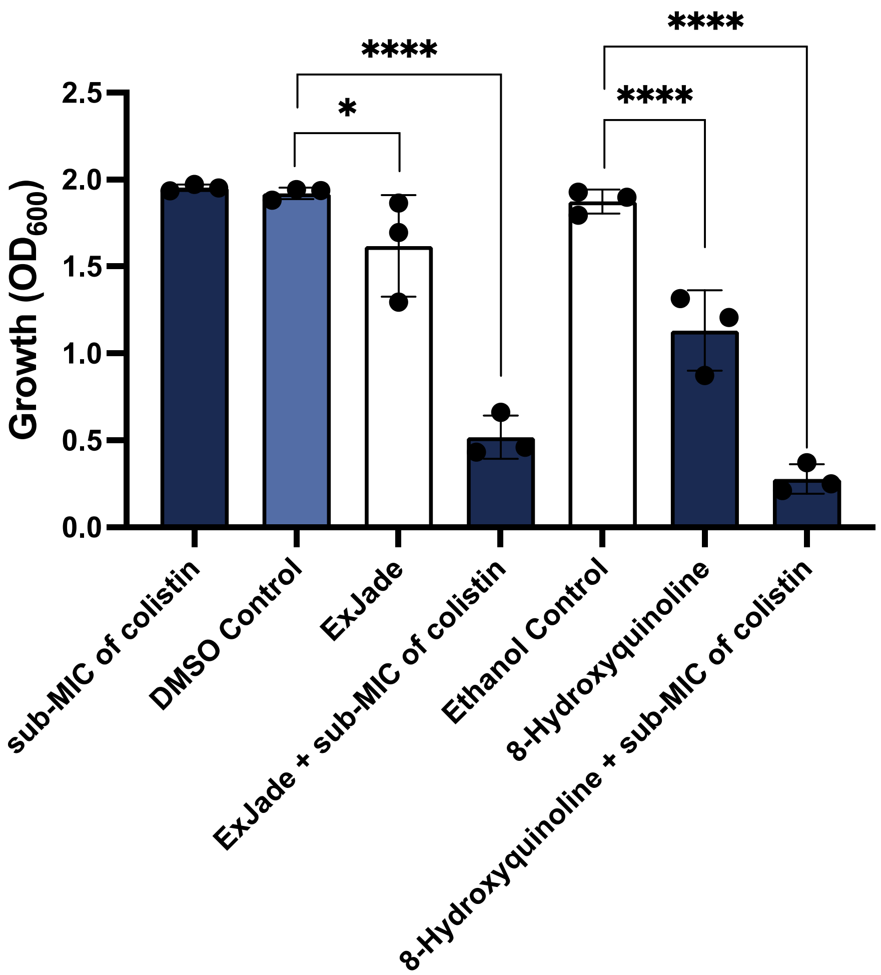

Supporting Information Figure S2. **ExJade and 8-Hydroxyquinoline at 25 µM potentiate the activity of colistin**. Colistin was used at sub-MIC amounts (1.22 µg/mL). Assays were carried out in biological triplicate (n=3), with three technical repeats. Analysis consists of two-way ANOVA between the treated samples and the DMSO or ethanol carrier controls, marked using asterisks (* = P ≤0.05, ** = P ≤0.01, ns = non-significant) with standard deviation error bars.

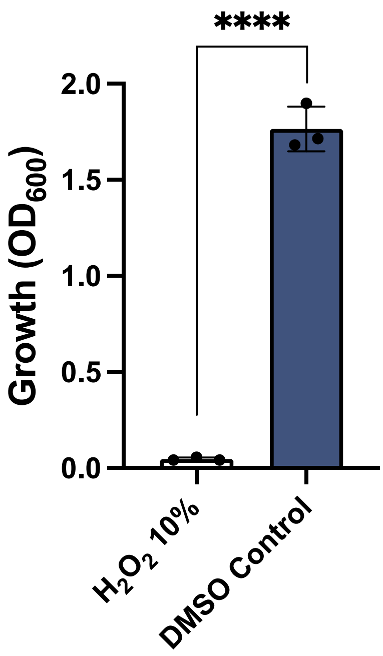

Supporting Information Figure S3. 10% v/v hydrogen peroxide is lethal to *A. baumannii*. The concentration of 10% v/v hydrogen peroxide (H_2O_2), used as a control condition for Figure 7A, is sufficient to inhibit bacterial growth. Assays were carried out in biological triplicate (n=3), with three technical repeats. Analysis consists of independent t-test between the treated samples and the DMSO carrier control, marked using asterisks (* = $p \leq 0.05$, ** = $p \leq 0.01$, ns = non-significant) with standard deviation error bars.

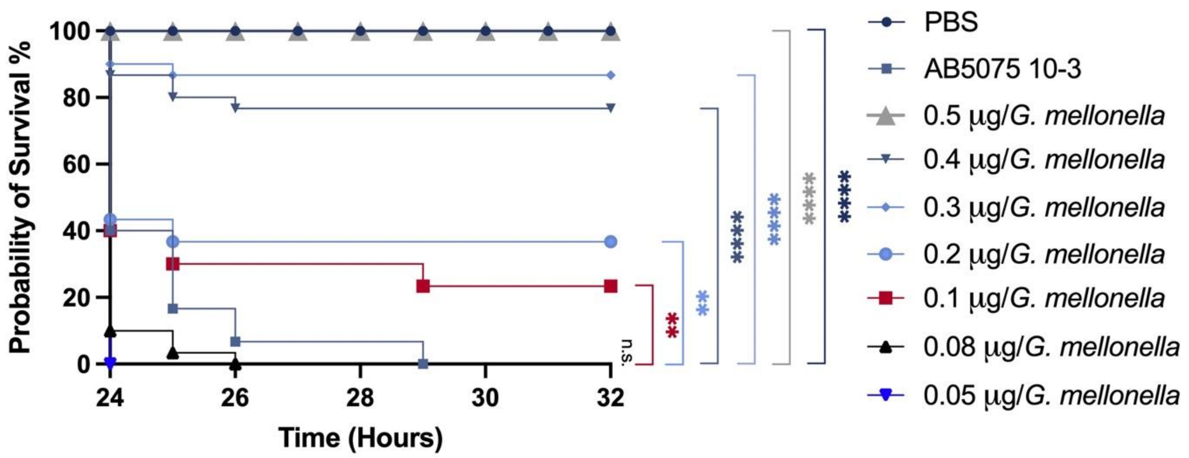

Supporting Information Figure S4. Efficacy of colistin against *A. baumannii* in the *Galleria mellonella* infection model. *G. mellonella* were injected with *A. baumannii* and treated with increasing concentrations of colistin. The lowest concentration of colistin allowing survival of the larvae was 0.1 µg/*G. mellonella*. As such, the amount of 0.08 µg of colistin per *G. mellonella*, which does not allow larval survival, was selected to be used in combination with kaempferol in subsequent experiments. Assays were carried out in biological triplicate per treatment group, with 30 *G. mellonella* used per group (N = 30). Analysis consisted of Log-rank (Mantel-Cox) test where no significant difference was observed between the AB5075 10-3 samples and the 0.08 µg/*G. mellonella* samples.

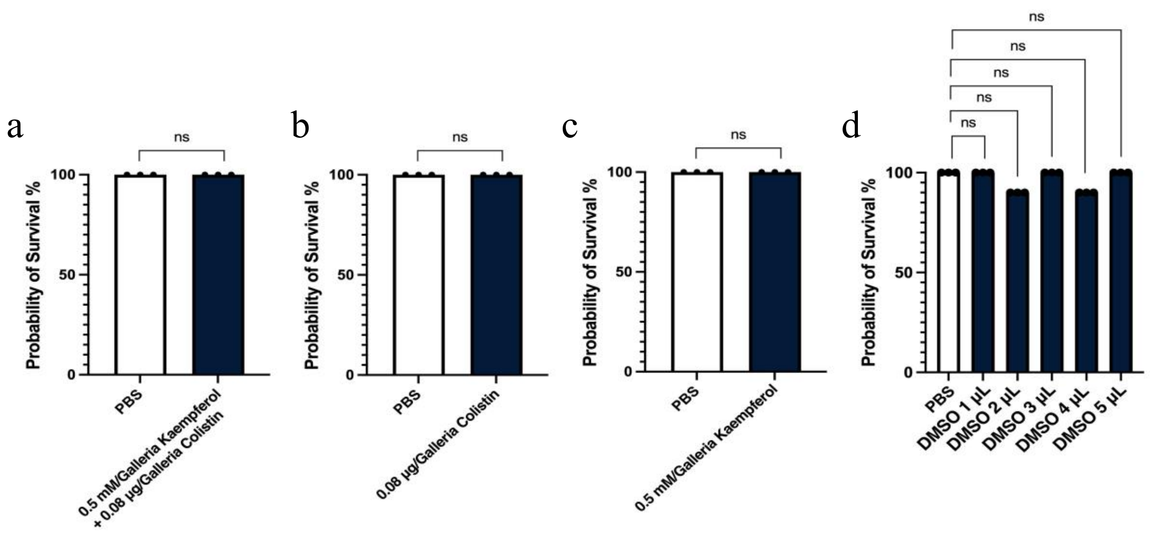

Supporting Information Figure S5. Cytotoxicity assays in the *Galleria mellonella* 32-hour infection model. **(A)** Sub-MIC amounts of colistin combined with kaempferol, **(B)** sub-MIC amounts of colistin, **(C)** kaempferol alone, and **(D)** the DMSO carrier control were tested in the *G. mellonella* model of infection. All tested conditions were demonstrated to not be cytotoxic to the larvae. Assays were carried out in biological triplicate per treatment group, with 30 *G. mellonella* used per group (N = 30). Analysis consisted of Log-rank (Mantel-Cox) test where no significant difference was seen between the samples and PBS control.

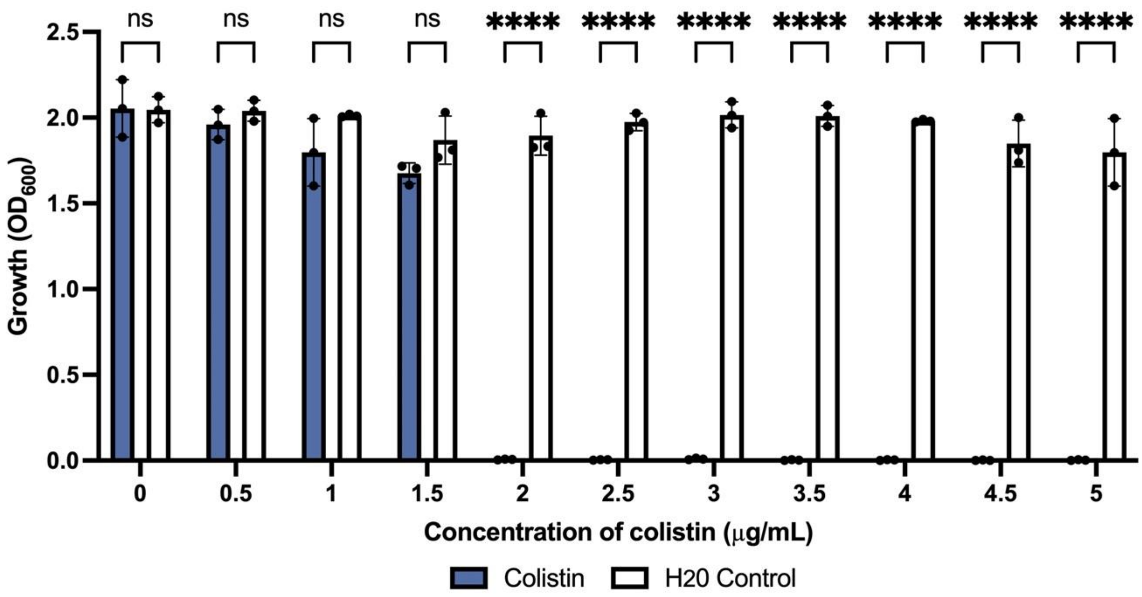

Supporting Information Figure S6. Minimum Inhibitory Concentration of Colistin against AB5075. Complete inhibition of visible growth of *A. baumannii* was observed with 2 μg/mL of colistin. Assays were carried out in biological triplicate (n=3), with three technical repeats. Statistical analysis consisted of two-way ANOVA between the treated samples and the growth control. Average values ± S. D. are represented. Significance is indicated as * = p ≤0.05, ** = p ≤0.01, *** = p ≤0.001, **** = p ≤0.0001.

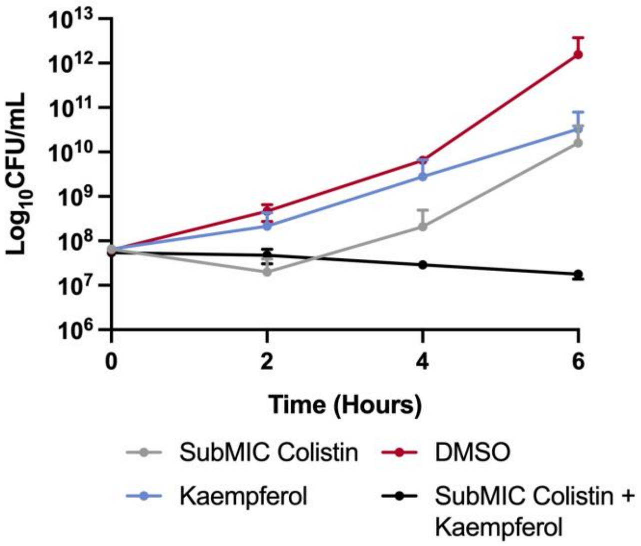

Supporting Information Figure S7. Time-kill curves of Combination treatment and its components against AB5075. A reduction in CFUs was observed when treated with the combination treatment only. Data is two biological replicates with three advanced technical replicates (n=2). Average values ± S. D. are represented.

Supporting Information Tables

Supplementary Table S1. Plasmids used in this study.

NAME	DESCRIPTION	SOURCE
pDM1	pDM1 vector (GenBank MN128719), p15A ori, Ptac promoter, MCS, Tet^R	Furniss RCD, et al. Breaking antimicrobial resistance by disrupting extracytoplasmic protein folding. *Elife*. 2022; **11**: e57974.
pDM1-mcr-1	*mcr-1* cloned into pDM1, Tet^R	Furniss RCD, et al. Breaking antimicrobial resistance by disrupting extracytoplasmic protein folding. *Elife*. 2022; **11**: e57974.
pRK2013	ColE1, tra+. Conjugative helper plasmid. Km^R	McCarthy lab collection, Figurski and Helinski, 1979
pTNS2	R6K replicon-based helper plasmid expressing the Tn7 transposase. Ap^R	McCarthy lab collection, Choi et al., 2005
pUC18T-miniTn7T-Tc-lacIq-Ptac	pUC18T derivative vector bearing a miniTn7T backbone including a Tc resistance marker and the *lacI^q-Ptac* expression system. Tc^R, Ap^R	McCarthy lab collection, de Dios *et al.*, 2022
pUC18T-miniTn7T-Tc-lacIq-Ptac::sodB	pUC18T derivative vector bearing a miniTn7T backbone including a Tc resistance marker and the *sodB* gene downstream the *lacI^q-Ptac* expression system. Tc^R, Ap^R	This work
pUC18T-miniTn7T-Tc-lacIq-Ptac::sodC	pUC18T derivative vector bearing a miniTn7T backbone including a Tc resistance marker and the *sodC* gene downstream the *lacI^q-Ptac* expression system. Tc^R, Ap^R	This work
pUC18T-miniTn7T-zeo-gfpmut3	pUC18T derivative vector bearing a miniTn7T backbone including a zeocin resistance	McCarthy lab collection, Choi & Schweizer, 2006

	marker and the *gfpmut3* coding regions to construct transcriptional fusions. Zeo^{R}, Ap^{R}	
pUC18T-miniTn7T-zeo-PsodB::gfpmut3	pUC18T derivative vector bearing a miniTn7T backbone including a *PsodB::gfpmut3* transcriptional fusion. ZeoR, Ap^{R}	This work
pUC18T-miniTn7T-zeo-PsodC::gfpmut3	pUC18T derivative vector bearing a miniTn7T backbone including a *PsodC::gfpmut3* transcriptional fusion. Zeo^{R}, Ap^{R}	This work

Supplementary Table S2. Oligonucleotides used in this study

**NAME**	**SEQUENCE**	**SOURCE**
AB5075-glmS fw	TTTGCTGATGAAAATAGTGG	de Dios *et al.*, 2022
Tn7R	CACAGCATAACTGGACTGATTTC	Kumar *et al.*, 2010
sodB fw RBS PstI	AAAAACTGCAGGAAAGAGGAGAATAGGAACATGACAACCATTAC	This work
sodB rv KpnI	AAAAAGGTACCTTATTTCTCTACACCAGCTGG	This work

sodB trx fw EcoRI	TTTTTGAATTCTGAAATTATTCAGTGGTGCG	This work
sodB trx rv BamHI	TTTTTGGATCCGGTTGTCATGTTCCTATTCC	This work
sodC fw RBS PstI	AAAAACTGCAGGAAAGAGGAGAAATCTATAATGCCAGTATTTAAT AAAATTGG	This work
sodC rv HindIII	AAAAAAAGCTTAAAGCACTTATTTGATTACACC	This work
sodC trx fw EcoRI	TTTTTGAATTCTATTCTACAACATGAAGGCCG	This work
sodC trx rv BamHI	TTTTTGGATCCAAATACTGGCATTATAGATATCC	This work

Supporting Information Methods

Iron complex formation assay

When flavonoids, such as kaempferol, form a complex with a metal, they exhibit a shift in their absorbance peak to higher wavelengths. To explore whether kaempferol forms a complex with iron and if this complex formation is specific for either Fe^{2+} or Fe^{3+}, we measured the absorption spectra in the 300-500 nm range of kaempferol mixed with either form of iron in different proportions, similarly to Catapano *et al*., 2017. Briefly, we mixed kaempferol with either Fe^{2+} or Fe^{3+} in different molecular proportions while keeping a total concentration of

both complexes of 0.1 mM in a total volume of 100 µl. Mixes were incubated at room temperature for 10 minutes. After that, the absorbance was measured in a Clariostar Plus plate reader (BMG LabTech). As a baseline control, mixtures with DMSO and water volumes equivalent to those of kaempferol and iron, respectively, were used. For measuring the kaempferol spectra, iron volumes were replaced by equivalent volumes of water. Three independent experiments were performed.

Time-kill assays

A. baumannii AB5075 overnight cultures were diluted in LB to an OD_{600} of 0.1. Treatments were set up in a 96-well plate and consisted of kaempferol (0.375 mM), the sub-MIC of colistin (1.22 µg/ml), the combination treatment of kaempferol and the sub-MIC of colistin and DMSO vehicle control. The plate was then incubated at 37 °C, 200 rpm, in a Clariostar Plus plate reader (BMG LabTech). An aliquot was taken at 0, 2, 4, 6 and 8 hours from each of the samples and diluted 1 in 10-fold, to 10^{-9}, and plated on LB agar. The plates were then incubated for 16 hours at 37 °C and bacteria colonies were counted.

Chelation Assay

A. baumannii AB5075 overnight cultures were diluted in LB to an OD_{600} of 0.1. Treatments were set up in a 96-well plate and consisted of kaempferol (0.375 mM), EDTA (100 µM), ExJade 25 µM or 8-Hydroxyquinoline 25 µM, the sub-MIC of colistin (1.22 µg/ml) and the combination treatment of kaempferol, EDTA, ExJade or 8-Hydroxyquinoline and the sub-MIC of colistin, with the respective DMSO vehicle controls. The plate was then incubated at 37 °C, 200 rpm, in a Clariostar Plus plate reader (BMG LabTech), where a OD_{600} reading was taken

every 10 minutes for 12 hours. The results represent the average of three technical replicates and three biological replicates ± S.D.

Hydrogen Peroxide Growth Assays

Overnight *A. baumannii* AB5075 cultures were diluted in CAMHB (pH 7.4) to get an OD_{600} of 0.1. Treatment consisted of hydrogen peroxide at 10% concentration, using the respective DMSO vehicle controls. The plate was then incubated at 37 °C, 200 rpm in a Clariostar Plus plate reader (BMG LabTech), where OD_{600} readings were taken every 10 minutes for 12 hours. The results represent the average of three technical replicates and three biological replicates ± S.D.
