# Supplementary material for: Disrupting iron homeostasis can potentiate colistin activity and overcome colistin resistance mechanisms in Gram-Negative Bacteria
Source: Commun Biol. 2023 Sep 13;6:937. doi: 10.1038/s42003-023-05302-2 (PMC10499790; doi:10.1038/s42003-023-05302-2)
Supplement: Supplementary file 3 — Description of Additional Supplementary Files [file 42003_2023_5302_MOESM3_ESM.pdf]

Description of Additional Supplementary Files

File name: Supplementary Data 1
Description: Transcriptomics.

File name: Supplementary Data 2
Description: Strains used in this study.

File name: Supplementary Data 3
Description: Numerical source data behind the graphs in the figures.
