# Supplementary material for: Disrupting iron homeostasis can potentiate colistin activity and overcome colistin resistance mechanisms in Gram-Negative Bacteria
Source: Commun Biol. 2023 Sep 13;6:937. doi: 10.1038/s42003-023-05302-2 (PMC10499790; doi:10.1038/s42003-023-05302-2)
Supplement: Supplementary file 7 — Reporting Summary [file 42003_2023_5302_MOESM7_ESM.pdf]

nature portfolio

Corresponding author(s): Ronan McCarthy
Last updated by author(s): Aug 24, 2023

Reporting Summary

Nature Portfolio wishes to improve the reproducibility of the work that we publish. This form provides structure for consistency and transparency in reporting. For further information on Nature Portfolio policies, see our Editorial Policies and the Editorial Policy Checklist.

Statistics

For all statistical analyses, confirm that the following items are present in the figure legend, table legend, main text, or Methods section.

n/a	Confirmed	
☐	☒	The exact sample size (*n*) for each experimental group/condition, given as a discrete number and unit of measurement
☐	☒	A statement on whether measurements were taken from distinct samples or whether the same sample was measured repeatedly
☐	☒	The statistical test(s) used AND whether they are one- or two-sided<br>*Only common tests should be described solely by name; describe more complex techniques in the Methods section.*
☒	☐	A description of all covariates tested
☐	☒	A description of any assumptions or corrections, such as tests of normality and adjustment for multiple comparisons
☐	☒	A full description of the statistical parameters including central tendency (e.g. means) or other basic estimates (e.g. regression coefficient) AND variation (e.g. standard deviation) or associated estimates of uncertainty (e.g. confidence intervals)
☐	☒	For null hypothesis testing, the test statistic (e.g. *F*, *t*, *r*) with confidence intervals, effect sizes, degrees of freedom and *P* value noted<br>*Give P values as exact values whenever suitable.*
☒	☐	For Bayesian analysis, information on the choice of priors and Markov chain Monte Carlo settings
☒	☐	For hierarchical and complex designs, identification of the appropriate level for tests and full reporting of outcomes
☒	☐	Estimates of effect sizes (e.g. Cohen's *d*, Pearson's *r*), indicating how they were calculated

Our web collection on statistics for biologists contains articles on many of the points above.

Software and code

Policy information about availability of computer code

Data collection	NA
Data analysis	Quality control and adapter trimming was performed with bclfastq. Read mapping was performed with HISAT. Differential expression analysis was performed using edgeR’s exact test for differences between two groups of negative-binomial counts with an estimated dispersion value of 0.1, using the A. baumannii AB5075-UW genome annotation as reference (Gallagher et al., 2015). The volcano plot was generated using R, by plotting the log fold change on the x axis and P value on the y axis. 117 genes were differentially expressed based on a log fold change ≥ 1 and P value < 0.05. A Gene Set Enrichment Analysis (GSEA) was performed using FUNAGE-Pro with the default parameters (de Jong et al., 2022).

For manuscripts utilizing custom algorithms or software that are central to the research but not yet described in published literature, software must be made available to editors and reviewers. We strongly encourage code deposition in a community repository (e.g. GitHub). See the Nature Portfolio guidelines for submitting code & software for further information.

Data

Policy information about availability of data

All manuscripts must include a data availability statement. This statement should provide the following information, where applicable:
- Accession codes, unique identifiers, or web links for publicly available datasets
- A description of any restrictions on data availability
- For clinical datasets or third party data, please ensure that the statement adheres to our policy

Transcriptomics datasets obtained through this work have been deposited at the Gene Expression Omnibus (GEO) repository of National Center for Biotechnology Information (NCBI) under the accession number GSE212989. Reviewers can access this dataset using the reviewer access token 'cpinoayslvivnar'.

Human research participants

Policy information about studies involving human research participants and Sex and Gender in Research.

Reporting on sex and gender	NA
Population characteristics	NA
Recruitment	NA
Ethics oversight	NA

Note that full information on the approval of the study protocol must also be provided in the manuscript.

Field-specific reporting

Please select the one below that is the best fit for your research. If you are not sure, read the appropriate sections before making your selection.

☒ Life sciences ☐ Behavioural & social sciences ☐ Ecological, evolutionary & environmental sciences

For a reference copy of the document with all sections, see nature.com/documents/nr-reporting-summary-flat.pdf

Life sciences study design

All studies must disclose on these points even when the disclosure is negative.

Sample size	10 larvae were used per group and each experiment was repeated on three separate occasions for a total of n=30 per group. Statistical Analysis consisted of Log-rank (Mantel-Cox) test with p<0.05 considered significant.
Data exclusions	Data was excluded if non infected controls displayed a morbidity of greater than 10% indicating a low health status of the larval colony.
Replication	Each assay was repeated on three independent occasions.
Randomization	Larvae were randomly assigned to experimental groups.
Blinding	Larvae were randomly assigned to experimental groups.

Reporting for specific materials, systems and methods

We require information from authors about some types of materials, experimental systems and methods used in many studies. Here, indicate whether each material, system or method listed is relevant to your study. If you are not sure if a list item applies to your research, read the appropriate section before selecting a response.

Materials & experimental systems

n/a	Involved in the study
☒	☐ Antibodies
☒	☐ Eukaryotic cell lines
☒	☐ Palaeontology and archaeology
☐	☒ Animals and other organisms
☒	☐ Clinical data
☒	☐ Dual use research of concern

Methods

n/a	Involved in the study
☒	☐ ChIP-seq
☒	☐ Flow cytometry
☒	☐ MRI-based neuroimaging

Animals and other research organisms

Policy information about studies involving animals; ARRIVE guidelines recommended for reporting animal research, and Sex and Gender in Research

Laboratory animals	Galleria mellonella
Wild animals	All in vivo experiments were conducted using G. mellonella (Live Foods Ltd) of 0.278 g average weight.
Reporting on sex	Larvae were randomly assigned to assigned to groups.
Field-collected samples	NA
Ethics oversight	No ethical approval required as Galleria mellonella are not covered by the Animals (Scientific Procedures) Act 1986

Note that full information on the approval of the study protocol must also be provided in the manuscript.
